# Diversity of epothilone producers among *Sorangium* strains in producer-positive soil habitats

**DOI:** 10.1111/1751-7915.12103

**Published:** 2013-12-06

**Authors:** Shu-guang Li, Lin Zhao, Kui Han, Peng-fei Li, Zhi-feng Li, Wei Hu, Hong Liu, Zhi-hong Wu, Yue-zhong Li

**Affiliations:** 1 State Key Laboratory of Microbial Technology, School of Life Science, Shandong UniversityJinan, 250100, China

## Abstract

Large-scale surveys show that the anti-tumour compounds known as epothilones are produced by only a small proportion of *Sorangium* strains, thereby greatly hampering the research and development of these valuable compounds. In this study, to investigate the niche diversity of epothilone-producing *Sorangium* strains, we re-surveyed four soil samples where epothilone producers were previously found. Compared with the < 2.5% positive strains collected from different places, epothilone producers comprised 25.0–75.0% of the *Sorangium* isolates in these four positive soil samples. These sympatric epothilone producers differed not only in their 16S rRNA gene sequences and morphologies but also in their production of epothilones and biosynthesis genes. A further exploration of 14 soil samples collected from a larger area around a positive site showed a similar high positive ratio of epothilone producers among the *Sorangium* isolates. The present results suggest that, in an area containing epothilone producers, the long-term genetic variations and refinements resulting from selective pressure form a large reservoir of epothilone-producing *Sorangium* strains with diverse genetic compositions.

## Introduction

**Figure 1:**
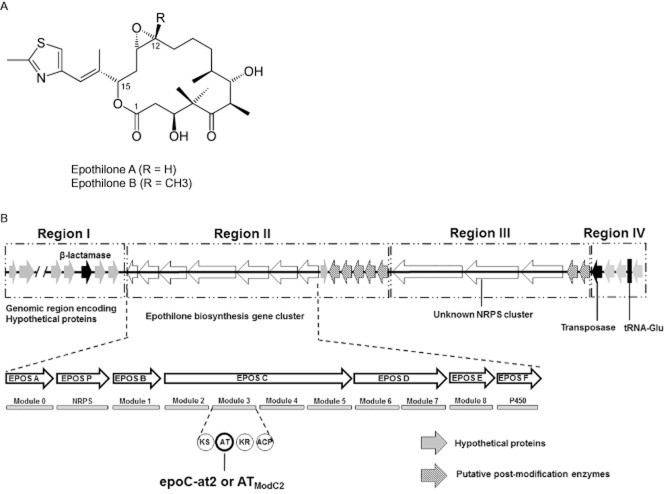
The molecular structures of the epothilone analogues A and B (A) and their biosynthetic gene cluster and its flanking region (B) in *S. cellulosum* So0157-2 according Han and colleagues (2013). The region in (B) has a length of 128 kb and can be divided into four independent subregions: Region I (length = 12 kb, GC% = 70%) encodes 12 hypothetical proteins and 1 β-lactamase, and none of the genes had synteny with the reported So ce56 genome. Region II (length = 61 kb, GC% = 69%) is responsible for epothilone biosynthesis and post-modification. Region III (length = 51 kb, GC% = 72%) is a non-ribosomal peptide synthetase (NRPS) gene cluster with no clear function, and all the genes arose from *Burkholderia*-sourced sequences. In region IV (length = 4 kb), the genes encoding a putative transposase and a tRNA gene (glutamic acid, Glu) are identified. ACP, acyl carrier protein; AT, acyltransferase; AT_ModC2_, the second acyltransferase domain of the *epoC* module; KR, β-ketoreductase; KS, β-ketoacyl synthase; NRPS, non-ribosomal peptide synthetases.

Myxobacteria are a special type of bacteria that adopt complicated multicellular social lifestyles (Whitworth, 2008). These bacteria are also known for their biosynthesis of diverse and novel secondary metabolites (Reichenbach, 2001). Among the different myxobacterial taxa, *Sorangium cellulosum* is an intriguing organism for drug-screening efforts because the secondary metabolic compounds discovered from *S. cellulosum* strains comprise up to 48.4% of the total metabolites obtained thus far from myxobacteria (Gerth *et al*., 2003). For example, epothilones (Fig. 1A), which act on cancer cells by mimicking the mechanism of Taxol, i.e. stabilizing microtubules (Bollag *et al*., 1995), are produced by *S. cellulosum* (Gerth *et al*., 1996). Biochemical, pharmacological and clinical studies have shown that epothilones are highly promising for cancer treatment (Reichenbach and Höfle, 2008). Some epothilones and their chemically modified derivatives are being used in clinical studies or trials (Larkin and Kaye, 2006), and one has been approved for clinical use by the U.S. Food and Drug Administration. However, in contrast to increasing progress in their applications, the production of epothilones in *Sorangium* strains is not optimal. *Sorangium* cells grow slowly, and they possess multiple antibiotic resistance capabilities, have abundant extracellular polysaccharides and exhibit a tendency to aggregate (Shimkets *et al*., 2006), all of which impede the isolation and cultivation of *Sorangium* strains as well as their genetic manipulation. For example, since the conjugation method was first developed in *S. cellulosum* in 1992 (Jaoua *et al*., 1992), genetic methods have improved (Pradella *et al*., 2002; Julien and Fehd, 2003; Kopp *et al*., 2004; Xia *et al*., 2008), but metabolic engineering in *Sorangium* remains inefficient. Many heterologous hosts have been employed for epothilone biosynthesis, including *Streptomyces coelicolor* (Tang *et al*., 2000), *S. venezuelae* (Park *et al*., 2008), *Escherichia coli* (Mutka *et al*., 2006), *Pseudomonas putida* (Fu *et al*., 2008) and *Myxococcus xanthus* (Julien and Shah, 2002; Lau *et al*., 2002; Fu *et al*., 2008; Oβwald *et al*., 2012), but epothilone production is very low in these heterologous hosts. The ratio of epothilone producers is also low among *Sorangium* strains (Gerth *et al*., 2001), and screening for producers of epothilones has to be based on large libraries of *Sorangium* strains. For example, taking advantage of their massive collection of *Sorangium* strains isolated from all over the world over the past decades, the former German Research Centre for Biotechnology (GBF) has identified dozens of epothilone producers (39 out of 1600) (Gerth *et al*., 2001; 2003). Therefore, searching for more epothilone producers is necessary not only to identify new strains with potentially suitable characteristics but also to obtain more strains for further genetic modification, as well as for studies of their biosynthetic mechanisms.

*Sorangium* is a genus of cellulolytic myxobacteria, and it is proposed to contain at least two species (Yan *et al*., 2003; Shimkets *et al*., 2006; Jiang *et al*., 2008). In classical isolation techniques, one or two clones of one myxobacterial species with obviously different morphological characteristics are normally isolated from a given soil sample (Dawid, 2000; Li *et al*., 2000; Shimkets *et al*., 2006). However, molecular surveys (Wu *et al*., 2005; Jiang *et al*., 2007) indicate that there are many myxobacterial 16S rRNA gene sequences with < 2% differences between them. For example, among the 85 randomly sequenced clones in one molecular ecological survey, five different sequences belonged to the *Sorangium* branch (Jiang *et al*., 2007). In other words, except of one or two isolates, many *Sorangium* linkages in soils may be ignored during normal large-scale isolation procedures. It is known that the growth of myxobacteria is cell density-dependent (Shimkets *et al*., 2006), and different strains of the same myxobacterial species normally separate to form respective colonies (Vos and Velicer, 2009), which suggest that separate colonies might represent different *Sorangium* strains. In fact, intraspecies diversity of *M. xanthus* was once reported in centimetre-scale soil samples (Vos and Velicer, 2008), which was later showed to be diverse in biosynthesis of secondary metabolites (Krug *et al*., 2008). In our previous studies, we performed a large-scale screen of more than 800 *Sorangium* strains isolated from 425 soil specimens collected in different places of China to identify the epothilone producers (Dong *et al*., 2004; Hu *et al*., 2004; Li *et al*., 2007), and the positive ratio was no more than 3%. In this study, to acquire more epothilone producers and investigate the diversity of *Sorangium* isolates in single soil samples, we re-surveyed four soil samples from which epothilone-producing strains or strains potentially possessing epothilone biosynthesis genes were discovered. Furthermore, the survey was extended to 14 specimens collected within approximately 10 km^2^ near one positive site.

## Results

### Diversity of *Sorangium* strains in soil niches

**Figure 2:**
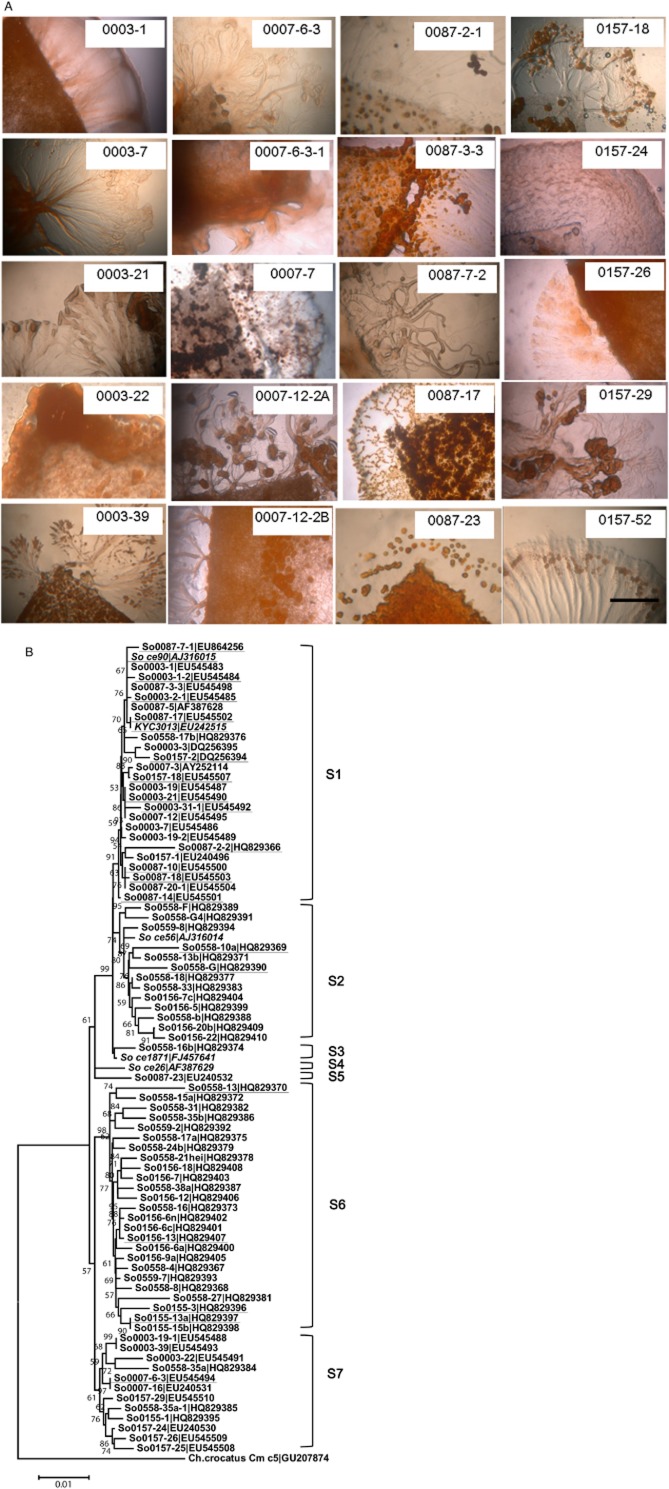
Morphological characteristics (A) and phylogenetic relationships (B) of selected *Sorangium* isolates. Strains in (A) were incubated on CNST medium for 2 weeks. Bar = 5 mm. These *Sorangium* representatives showed different morphological characteristics, such as swarm shapes, structure and colour of fruiting bodies and sporangioles. The phylogenetic tree in (B) was constructed using 16S rRNA gene complete sequences. *Chondromyces crocatus* Cm c5 (GenBank accession number GU207874) was used as the root. *Sorangium* strains So ce26 (AF387629), So ce56 (AJ316014), So ce1871 (FJ457641) and the epothilone-producing strains, So ce90 (AJ316015) and KYC3013 (EU242515), were used as references (shown in italics). The epothilone producers are underlined for easy tracking. The bar is equivalent to one nucleotide change per 100 bp, The bootstrap support is from 1000 replicates. S1–S6 represent *Sorangium* subgroups 1–6 respectively.

In our previous screening studies of hundreds of *Sorangium* strains (Dong *et al*., 2004; Hu *et al*., 2004), three epothilone producers (designated So0003-3, So0007-3 and So0157-2) were obtained from three soil samples (soil ID numbers 0003, 0007 and 0157). An additional strain, So0087-5, from the 0087 soil sample, did not produce epothilones, but the requisite biosynthesis genes might be present, as an almost identical ketoacyl synthase (KS) domain (704 bp) for the biosynthesis of epothilones was detected in So0087-5 (Identities = 98%, Positives = 98%) (Li *et al*., 2007). These four soil samples (Table 1) were regarded as positive soil samples and were re-surveyed for niche diversity of epothilone producers in this study. We picked and purified 8 to more than 20 separated *Sorangium* colonies from the four soil samples, depending on the number of colonies that appeared on the isolation medium. These *Sorangium* isolates grew well on mineral medium with filter paper as the only carbon source (CNST medium). The isolates from the same soil samples had either similar or different morphological characteristics (some representative *Sorangium* morphologies from each sample are shown in Fig. 2A). It was noted that some isolates, such as So0003–22, So0007-6-3-1 and So0157-24, could barely form fruiting body structures on CNST medium. Isolation of these non-fruiting *Sorangium* strains was based on the characteristics of *Sorangium* swarms. Phylogenetic analysis demonstrated that most of these separate *Sorangium* clones from one soil sample differed in their 16S rRNA gene sequences (Fig. 2B). The sympatric *Sorangium* strains often clustered into single phylogenetic groups but also included separate members. For example, nine of the 12 sequenced 0003 isolates were in group S1, and three were in group S7; whereas except for one in group S5, 9 of the 10 sequenced 0087 isolates were in group S1. However, these isolates showed significant phylogenetic differences. The largest phylogenetic distance between sympatric isolates was between So0003–31-1 and So0003–22 and was approximately 2.2%, which is similar to the phylogenetic distance observed for different soil samples (Yan *et al*., 2003). These results indicated that *Sorangium* strains were morphologically and phylogenetically diverse in single soil samples.

**Figure:**
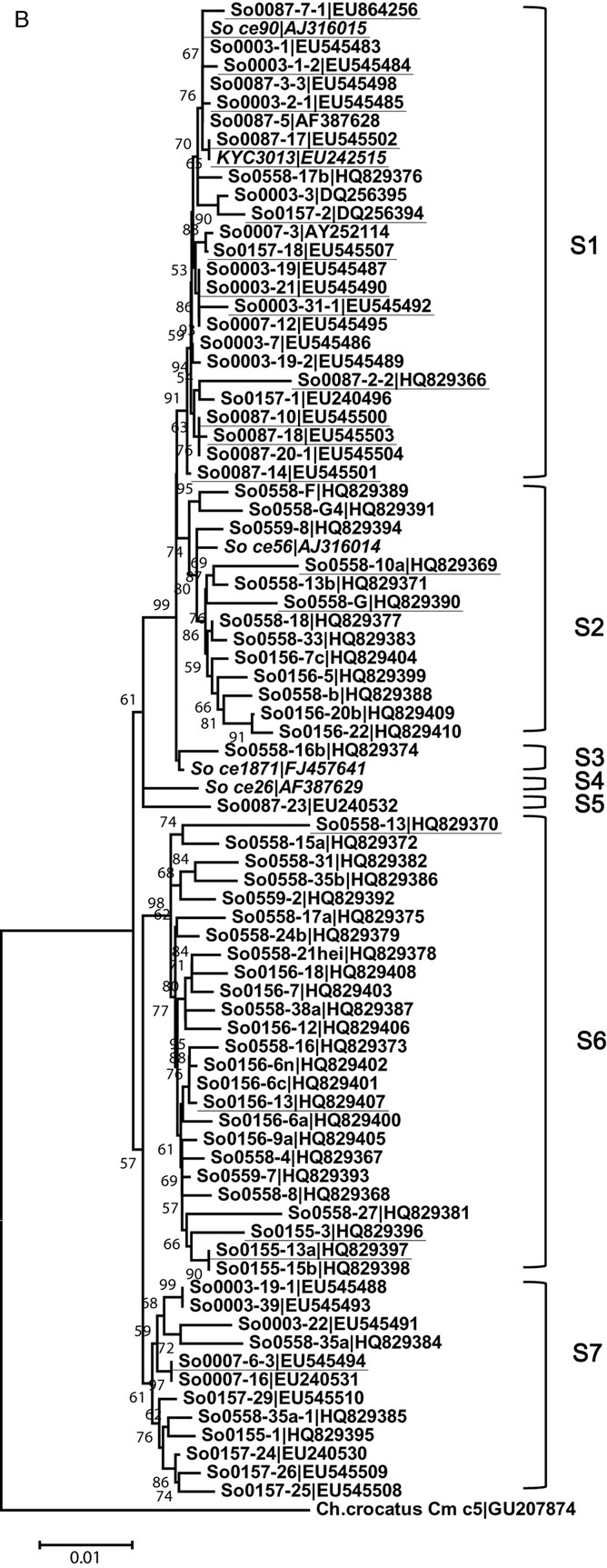


**Table 1:** Information of the soil samples analysed in this study and the positive ratios of epothilone producers among the *Sorangium* isolates

Soil samples*	Soil information	Isolation number	Detected number	Epothilone producer	Positive ratio
0003	A wheat field in Jiangsu Province, collected in February, 1996	13	13	4	30.8%
0007	A vegetable field in Jiangsu Province, collected in February, 1996	8	8	3	37.5%
0087	A paddy field, in Fujian Province, collected in August, 1996	24	24	18	75.0%
0157	An alkaline soil in Yunnan Province, collected in September, 2002	12	8	2	25.0%
0155	An alkaline soil near the 0157 site, collected in September, 2002	14	14	6	42.9%
0156	An alkaline soil near the 0157 site, collected in September, 2002	22	2	1	50.0%
0558	An alkaline lake mud near the 0157 site, collected in August, 2008	73	51	13	25.5%
0559	An alkaline lake mud near the 0157 site, collected in August, 2008	10	10	1	10.0%
Total		176	130	48	36.9%

* Other assayed 10 soils near the 0157 site: nine produced no *Sorangium* isolates (0146, 0147, 0148, 0158, 0555, 0556, 0557, 0560 and 0561); while one had six isolates but none was epothilone producers (0145).

### Production of epothilones in the *Sorangium* isolates

As determined by high-pressure liquid chromatography (HPLC)-mass spectrometry (MS), the positive ratio of epothilone producers ranged from 25.0% to 75.0% of the *Sorangium* isolates in the four soil samples (Table 1; the production of epothilone A in different strains is listed in Table 2; some HPLC and MS values are shown in Supplementary material S1). The positive ratio for the production of epothilones (27/53) in these four positive samples was significantly higher than the previous reports (39/1600) from unselected soil samples (Gerth *et al*., 2001; 2003) (*P* < 0.001, Fisher's exact test). We checked some soil samples, i.e. 0081, 0085, 0133, 0139, 0184 and a campus garden soil sample in Shandong University, from which epothilone-producing *Sorangium* strains were not found (Dong *et al*., 2004; Hu *et al*., 2004; Li *et al*., 2007). In the resurveys, 5 to more than 10 separated *Sorangium* colonies were isolated and purified from these six ‘negative’ soil samples, but none of them were epothilone producers (data not shown), which is consistent with our previous screen. The results suggested that epothilone producers were normally restricted in some places, instead of being ubiquitous in soil. However, considering there were also high ratios of non-epothilone producers in those ‘positive’ soil samples, screening on two or three isolates probably missed epothilone producers in a soil sample. For example, although our screening for the production of epothilones was negative with two strains isolated from the 0087 soil sample (Dong *et al*., 2004; Hu *et al*., 2004), many *Sorangium* isolates from the soil yielded epothilones. These results suggested that the previously reported positive ratio was an underestimation of the epothilone producers among the *Sorangium* strains isolated from different samples.

**Table 2:** Production of epothilone A by some *Sorangium* isolates on solid CNST medium after 2-week fermentation

Isolate	Epothilone A (mg l^−1^)	Isolate	Epothilone A (mg l^−1^)
So0003-1-2	0.53 ± 0.02	So0087-26	17.09 ± 1.34
So0003-2-1	0.34 ± 0.02	So0157-2	2.47 ± 0.09
So0003–21	4.66 ± 0.11	So0157-18	1.58 ± 0.04
So0003–31-1	13.78 ± 2.27	So0155-3	0.01
So0007-6-3	4.50 ± 1.08	So0155-4a	0.01
So0007–12-2A	15.71 ± 2.30	So0155-9	0.01
So0007–12-2B	15.08 ± 1.87	So0155-13	0.01
So0087-2-1	4.68 ± 0.89	So0155-13a	0.01
So0087-2-2	1.94 ± 0.24	So0155-15	< 0.01
So0087-2-3	< 0.01	So0156-13	0.01
So0087-3-2	5.38 ± 0.16	So0558-8a	0.01
So0087-7-1	9.39 ± 0.38	So0558-10a	0.02 ± 0.01
So0087-7-2-1	16.25 ± 3.32	So0558-11b	0.01
So0087-7-2-2	11.97 ± 1.80	So0558-13	0.01
So0087-10	9.18 ± 2.15	So0558-16a	< 0.01
So0087-13	3.16 ± 0.62	So0558-17	< 0.01
So0087-13-2	10.19 ± 2.20	So0558-23	0.13 ± 0.04
So0087-14	17.05 ± 1.33	So0558-29b	0.01
So0087-15	20.43 ± 2.51	So0558-31a	0.01
So0087-15-2	6.25 ± 1.09	So0558-G	2.42 ± 0.83
So0087-16	8.89 ± 1.07	So0558-G1	0.52 ± 0.18
So0087-17	12.88 ± 1.25	So0558-G2	0.75 ± 0.26
So0087-18	0.54 ± 0.02	So0558-G3	1.54 ± 0.53
So0087-20-2	6.87 ± 0.50	So0559-4	0.01

* For strains that had average productions ≤ 0.01, the standard deviations were even smaller, and weren't shown in the table.

### Biosynthesis genes for epothilones

**Figure 3:**
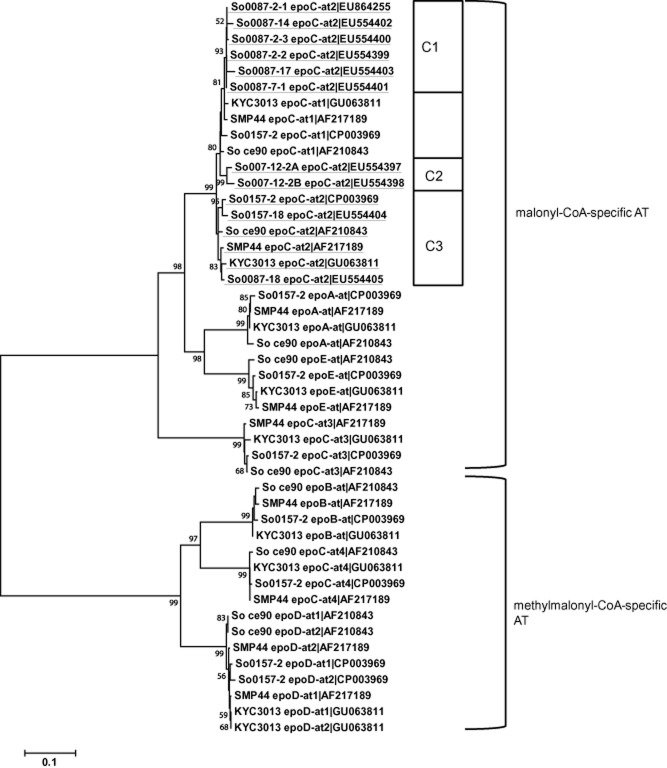
Phylogenetic analysis of the protein sequences of AT domains in epothilone biosynthesis clusters. AT_ModC2_ are underlined for easy tracking. The bar is equivalent to 10 amino acids change per 100 bp. The bootstrap support is from 1000 replicates, and only values greater than 50 are shown. C1–C3 represent three subgroups of AT_ModC2_.

Epothilones are biosynthesized via a seven modules of type I modular polyketide synthase (PKS) mixed with a non-ribosomal peptide synthetase (Julien *et al*., 2000; Molnár *et al*., 2000) (Fig. 1B). To determine the differences of epothilone biosynthesis genes between multiple producers, we sequenced AT_ModC2_ in some of the epothilone-producing *Sorangium* isolates. Acyltransferase (AT) domains are fundamental elements of type I PKSs (Cheng *et al*., 2003; Ginolhac *et al*., 2004; Jenke-Kodama *et al*., 2005). Phylogenetic analysis indicated that the epothilone AT domains were distinct from those of other myxobacteria and non-myxobacteria (Fig. S1). The AT protein sequences of all reported epothilone biosynthesis cluster in So0157-2 (Han *et al*., 2013), So ce90 (Molnár *et al*., 2000), SMP44 (Tang *et al*., 2000) and KYC3013 (Hyun *et al*., 2008) were phylogenetically analysed (Fig. 3), which showed that the epothilone ATs were sorted into two groups, malonyl-coenzyme A (CoA) specific and methylmalonyl-CoA specific. The distances of AT domains in the same modules were < 3.2%, with an average value of 1.3 ± 0.8%. It is noticed that the second AT domain of the *epoC* module (AT_ModC2_), which is supposed to indiscriminately accept malonyl-CoA for epothilone A or methylmalonyl-CoA for epothilone B (Gerth *et al*., 2000; Molnár *et al*., 2000; Yadav *et al*., 2003; Petković *et al*., 2008), was also located in the malonyl-CoA-specific group.

Similar to that of the 16S rRNA gene sequences, the amino acid sequences of AT_ModC2_ of sympatric *Sorangium* isolates were often located in the same branches but also included separate members (Fig. 3). For example, among the seven sequenced AT_ModC2_ domain from sample 0087, six were in the same branch (C1), whereas one was rather distant from the others (So0087-18 in C3). The largest distance between different AT_ModC2_ was approximately 5.8%, between So0087-17 and So0157-18. These results suggested that the biosynthetic genes for epothilones might be different in those separate epothilone *Sorangium* producers obtained from single soil samples.

### Screening for epothilone producers around a positive site

In the course of evolution, bacteria distributed globally; therefore, epothilone producers could be found on different continents (Bollag *et al*., 1995; Gerth *et al*., 1996; Dong *et al*., 2004; Hyun *et al*., 2008). To investigate the spreading and distribution capacity of epothilone-producing *Sorangium* strains, 14 soil specimens collected from different locations near the 0157 site were further assayed. The sampling locales were over approximately 10 km^2^, and the samples were all alkaline, similar to that of the 0157 soil sample. *Sorangium* strains were found in five samples, including 0145, 0155, 0156, 0558 and 0559, and the appearance ratio of *Sorangium* organisms in this region was significantly higher (35.7%, 5/14) than that described in the previous report (5.2%, 73/1398) (Dawid, 2000) (*P* < 0.001, Fisher's exact test). Six to seventy-three separate *Sorangium* clones were isolated from these five soil samples, depending on their appearance on isolation plates. Consistent with the alkaline characteristics of the soils, most of the *Sorangium* isolates preferred to grow at pH 9.0 (alkali-tolerant). Epothilone producers were found in four of the soil samples, and the percent of epothilone producers among the detected *Sorangium* isolates was 27.3% (21 out of 77) (**Table** 1; the production abilities of the epothilone producers are listed in **Table** 2), which had no significant difference with that in the initial positive 0157 sample (*P* = 1000 > 0.05, Fisher's exact test). Similarly, phylogenetic analysis of the 16S rRNA gene sequences of some new isolates showed that they differed, normally located in the same branches in the phylogenetic tree, but they also included some separate strains in different branches (Fig. 2B). The largest phylogenetic distance between isolates from the same soil sample was approximately 3.1%, between So0558-10a and So0558-13. These results suggest that epothilone producers frequently appear in a large area around the established positive site (0157 sample) where they are able to live.

## Discussion

As a result of both genetic variation and environmental selection, bacteria have evolved into the most diversified taxonomic group on Earth (Giovannoni and Stingl, 2005). This biodiversity, derived during evolution, may also involve particular metabolic pathways of a single species, such as the biosynthesis of epothilones in *Sorangium* strains. The biosynthetic enzymes for epothilones have been determined to be encoded by a chromosome-associated gene cluster in *Sorangium* cells (Julien *et al*., 2000; Molnár *et al*., 2000). In fact, except for the pMF1 plasmid from *M. fulvus* 124B02 (Zhao *et al*., 2008), there has been no report of extrachromosomal genetic materials in myxobacteria up to now. The biosynthesis gene cluster for epothilones has been reported in several *Sorangium* producers (Bollag *et al*., 1995; Gerth *et al*., 1996; Gong *et al*., 2007; Hyun *et al*., 2008), but the organization of the gene cluster on chromosomes is still not known. Analysing the genome sequence of one of our epothilone producers, *S. cellulosum* So0157-2 (GenBank accession number CP003969) (Han *et al*., 2013), suggested that a 128 kb region (Fig. 1B) including the epothilone gene cluster possibly arose from horizontal gene transfer. First, the whole 128 kb region has no synteny with the *S. cellulosum* So ce56 genome (Schneiker *et al*., 2007) (Fig. S2), and all the genes in region III are predicted to be from *Burkhoderia*-sourced sequences. Second, tRNA and putative transposase genes are present near the end of region III (Chien *et al*., 2004). Third, an antibiotic gene (β-lactamase), often the element of integrons contained in some transposons (Levy and Marshall, 2004), is present in region I. We compared the sequences of the complete epothilone gene clusters in strains So0157-2, So ce90 and SMP44, which were isolated from different continents. The results revealed the high similarity among these three gene clusters with an average 98.5% identity, even in the non-coding intergenic spacer sequences (Fig. S3), suggesting that the biosynthesis genes for epothilones are highly conserved and origination-monophyletic, even though they are usually totally absent in non-producers (Schneiker *et al*., 2007).

The lack of diversified producers and efficient genetic performance systems is a serious limiting factor for the genetic engineering of epothilone producers and the industrialization of epothilone production. Furthermore, because of social characteristics of *Sorangium* strains, difficulties in their isolation and cultivation make this approach hard to achieve. In the current study, resurveying epothilone-positive soil samples using classical colony-based isolation techniques, we determined that *Sorangium* strains in small habitats were a mixture of different strains, not only based on 16S rRNA gene sequences and morphologies but also the production abilities of epothilones and their biosynthesis genes. Such an intraspecies diversity of sympatric *Sorangium* strains is potentially useful for various applications. For example, the strains synthesizing valuable metabolites are regarded as potential industrial producers; however, the yields of metabolites in these fresh isolates are usually too low to be industrialized. Normally, after the first round of isolation, the diverse producing strains that remain in the soil are ignored and are not further re-mined. Instead, many efforts have been made to improve the initial yield by metabolic engineering and fermentation optimization in laboratory. However, the ill-defined backgrounds of these organisms and the lack of efficient genetic manipulation systems make metabolic engineering difficult to achieve or time-consuming, especially in undomesticated micro-organisms, such as myxobacteria. Here, we demonstrated that compared with the low positive ratios (< 2.5%) in different soil samples, epothilone producers constituted the major proportion (25–75%) of the *Sorangium* isolates in positive soils. An analysis of epothilone production and the 16S rRNA gene sequences revealed that epothilone producers could be distributed in different branches of a *Sorangium* phylogenetic tree, even though the positive ratio of epothilone producers in group S1 is larger than other groups (Fig. 2B). Because of the marked differences in the ability to produce epothilones and the fine differences in the biosynthesis genes (demonstrated using AT_ModC2_), we suggest that the sympatric epothilone-producing *Sorangium* strains are highly diverse. Furthermore, epothilone-producing *Sorangium* cells were able to spread, acclimate and inhabit neighbouring locales where, if they remained viable, they formed a large reservoir of diversified epothilone producers. In billions of years of natural evolution, micro-organisms may generate many changes within a metabolic pathway, and such changes are further refined by selection. The promiscuous genetic composition of epothilone-producing *Sorangium* strains in positive areas provides many opportunities for the selection of promising candidates with the desired characteristics. For example, in the producers isolated from the 0087 soil sample, the yield of epothilone A changed from < 0.01 mg l^−1^ of So0087-2-3 to 20.43 mg l^−1^ of So0087-15, increasing more than 2000 times. Although the production of epothilones was affected by many factors, such as the growth rate of the cells on solid fermentation medium, the marked variation showed marked differences in the production ability of epothilones by different strains. It is thus an alternative and clearly an efficient and easy way to select strains with desirable characteristics, especially for those micro-organisms that are not easily genetically manipulated in the laboratory.

## Experimental procedures

### Isolation of *Sorangium* clones

Four soil samples from which epothilone-producing strains or strains possessing the epothilone biosynthesis genes had been discovered (Dong *et al*., 2004; Hu *et al*., 2004; Li *et al*., 2007) were collected from different regions and environments in China (Table 1). All of the soil samples were collected from a depth of 10–15 cm under the soil surface. After collection, the soils were air-dried immediately and stored at room temperature. CNST medium (Yan *et al*., 2003) was used to isolate the cellulolytic myxobacterium *Sorangium* strains. Before autoclaving, the pH of the medium was adjusted to 7.0–7.2. Because the 0157 soil sample was alkaline, the enrichment medium was prepared at two pH values, 7.0 and 9.0, after autoclaving. Small pieces (approximately 1 by 1 cm) of sterilized filter paper were placed on the medium surface as the carbon source. Soil samples were spread over the paper and incubated at 30°C. To allow for the appearance of discrete *Sorangium* clones on the filter paper, the soil was ground to avoid any clots and then spread in a thin layer. Ten plates were used for the isolation of *Sorangium* clones from each soil sample. Growth on the enriching plates was observed under a dissecting microscope. Clones with *Sorangium* phenotypes, including fruiting body structures and swarms, were carefully isolated with an inoculating needle and transferred to WAT plates smeared with autoclaved *E. coli* or VY/2 plates (Shimkets *et al*., 2006) for further purification using standard techniques. The purified isolates were cultured routinely on CNST medium at 30°C and morphologically and phylogenetically characterized according to previously described methods (Yan *et al*., 2003).

### Cultivation for the production of epothilones

Because most of the newly isolated *S. cellulosum* clones grew poorly in liquid, the production of epothilones was measured on solid CNST medium in plates (Gong *et al*., 2007). To prepare the inocula for cultivation, the cells were first inoculated onto filter paper placed on CNST plates and incubated at 30°C for 4–5 days (Hou *et al*., 2006). The cells and the destroyed filter paper were then scraped and collected separately from each plate using an inoculation shovel. The cells were suspended and gently homogenized with glass beads (3 mm in diameter) in sterilized water, centrifuged (5000 r.p.m., 5 min, 4°C) and resuspended in sterile water at approximately 1 × 10^7^ cells ml^−1^ (Gong *et al*., 2007). An aliquot of 2 ml of the cell suspensions was spread over an entire filter paper (70 mm in diameter) on CNST plate. After 4–5 days of incubation at 30°C for cellular growth, Amberlite XAD-16 resin beads (Rohm and Haas, Philadelphia, PA, USA) were spread over the colonies to absorb the epothilone products to avoid their feedback inhibition on the production (Gerth *et al*., 1996). The cultures were incubated for an additional 9–10 days. Epothilone production was measured in triplicate and was calculated as the yield of epothilone A divided by the volume of the total medium (mg l^−1^).

### Detection and identification of epothilones

The resin beads that were spread over the clones were harvested, washed with distilled water, air-dried and extracted with 5 ml of methanol. The extracts were then dried *in vacuo* at 40°C and stored at −20°C. For HPLC-MS analysis, samples were redissolved in 100 μl of methanol. A 10 μl aliquot was injected into a Surveyor HPLC (Thermo Finnigan, Pittsburgh, PA, USA) interfaced with a Finnigan MSQ classic quadrupole mass spectrometer (ESI-positive) (Thermo Finnigan). The analysis was performed on a Shim-pack MRC-ODS analytical reverse phase column (4.6 mm × 250 mm, 4.60 μm; Shimadzu, Tokyo, Japan) at a column temperature of 28°C with a mobile phase of 60% methanol (HPLC grade, Merck, Darmstadt, Germany) and 40% buffer (0.2% A.P. acetate acid/18 MΩ Millipore water) at a flow rate of 1.0 ml min^−1^. The production of epothilone A, eluted at 20 min with baseline resolution, was detected at 249 nm, and the titre was quantified based on a standard curve generated using purified epothilone A (Gong *et al*., 2007). The MS analysis was performed under the following conditions: ESI-positive, probe temperature of 450°C, cone voltage of 75 V, full scan mass range from 300 to 900 amu and SIM scan at 494 [M + H]^+^ for epothilone A.

### DNA extraction and polymerase chain reaction (PCR) amplification

DNA extraction and PCR amplification were performed as described previously (Li *et al*., 2007). The 16S rRNA gene sequences were amplified with primer pair 27F (5'-AGTTTGATCCTGGCTCAG-3') and 1492R (5'-TACCTTGTTACGACTT-3'). After constructed in the sequencing plasmid pMD 19-T Vector (Takara Biotechnology, Dalian, China), the PCR products were sequenced in both directions (Yan *et al*., 2003). To amplify the second AT domain in the *epoC* module (AT_ModC2_) of the epothilone biosynthesis genes, a nested PCR technique was employed to avoid false priming. The first primer set was 5'-ACGTCGATTTCGTGGAATGC-3' and 5'-AGTGGACGCATGACGCTGAC-3', locating in the upstream β-KS and downstream β-ketoreductase domains (Fig. 1B). The product is 2.7 kb, containing the AT_ModC2_ domain. The primer set for the second round was 5'-CTGCGCGAGCACCTGGACATGC-3' and 5'-GCTGCCGCTGCCACGGATAGGT-3', targeting a 1.5 kb AT_ModC2_-containing product within the product of the first amplification round.

### Phylogenetic analysis

The 16S rRNA gene sequences from different *Sorangium* strains and the second AT domain in the *epoC* module (AT_ModC2_) from different epothilone producer strains were analysed using the Neighbor-Joining programme in MEGA version 5.05 (Biodesign Institute, AZ, USA) (Tamura *et al*., 2011).

### Screening for epothilone producers around a positive site

To investigate the spreading and distribution abilities of epothilone-producing *Sorangium* strains, 14 soil specimens (Table 1) were collected from different places around the 0157 site. The sampling locales were within approximately 10 km^2^, and the samples were all alkaline, similar to that of the 0157 soil. *Sorangium* strains were isolated, and epothilone production was detected as described above.

### Accession numbers in GenBank

The nucleotide sequence data are available at GenBank under the following accession numbers: AF387628 (So0087-5), AY252114 (So0007-3), DQ256394 (So0157-2), DQ256395 (So0003-3), EU240496 (So0157-1), EU240530 (So0157-24), EU240531 (So0007–16), EU240532 (So0087-23), EU240533 (So0157-52), EU545483 to EU545510, EU864256 (So0087-7-1), HQ829366 to HQ829379, HQ829381 to HQ829410 for 16S rRNA genes, EU554397 to EU554406 and EU864255 for the second AT domain in the *epoC* module (AT_ModC2_) for epothilone biosynthesis in *Sorangium* strains. The GenBank accession number of the *S. cellulosum* So0157-2 genome is CP003969.

## Supporting information

Supplementary material S1 The HPLC and MS figures of purified epothilone A standard (A) and epothilone A produced by some strains (B, C, D).

Fig S1 Phylogenetic analysis of the protein sequences of AT domains retrieved from sequenced myxobacterial genomes. Some non-myxobacterial AT sequences were taken as reference. AT domains from epothilone biosynthesis clusters were compressed into two red triangles for easy tracking. The trees were constructed in a cycle using the MEGA 5.05 programme. Bootstrap support was based on 1000 replicates. One unit along the bar is equivalent to 10 nucleotides change per 100 bp. The outermost cycle was added after construction. Beside *S. cellulosum* So0157-2 and So ce56, other myxobacterial strains used in AT domains extracting are *Anaeromyxobacter dehalogenans* 2CP-1, *Anaeromyxobacter dehalogenans* 2CP-C, *Anaeromyxobacter* sp. Fw109, *Anaeromyxobacter* sp. K, *Haliangium* *ochraceum* dsm14365, *Myxococcus fulvus* HW-1, *Myxococcus xanthus* DK1622, *Stigmatella aurantiaca* dw4/3. The two numbers linked by ‘...’; showed the positions of the AT sequences in the genome.

Fig S2 The upstream and downstream region of the ‘128 kb length epothilone gene cluster and its flanking region’ in So 0157-2, when compared with So ce56. The biosynthesis gene cluster for epothilones and its flanking regions were extracted from the completely sequenced genome of the So0157-2 strain (Han *et al*., 2013). The open reading frames (ORFs) were predicted using Glimmer 3.02 (Delcher *et al*., 2007) and GeneMark.hmm (Borodovsky and Lomsadze, 2011). All ORFs larger than 50 amino acid residues were blasted against the NR database of the NCBI with a cut-off of 1 E–5. The GC content was calculated by GEECEE of the EMBOSS programme (Rice *et al*., 2000).

Fig S3 Similarity of the epothilone biosynthesis gene clusters (about 56 kb length) from three different Sorangium strains So ce 90 (AF210843), SMP44 (AF217189) and So0157-2 (CP003969). Each domain of the three whole gene clusters was homologous with an average of 98.5% identity, indicated by colourful histograms. The similarities of the intergenic spaces were also shown by grey histograms.
