# Supplementary material for: Diversity of epothilone producers among Sorangium strains in producer-positive soil habitats
Source: Microb Biotechnol. 2013 Dec 6;7(2):130–41. doi: 10.1111/1751-7915.12103 (PMC3937717; doi:10.1111/1751-7915.12103)
Supplement: Supplementary material S1 — The HPLC and MS figures of purified epothilone A standard (A) and epothilone A produced by some strains (B, C, D). [file mbt20007-0130-sd1.ppt]

## Slide 1
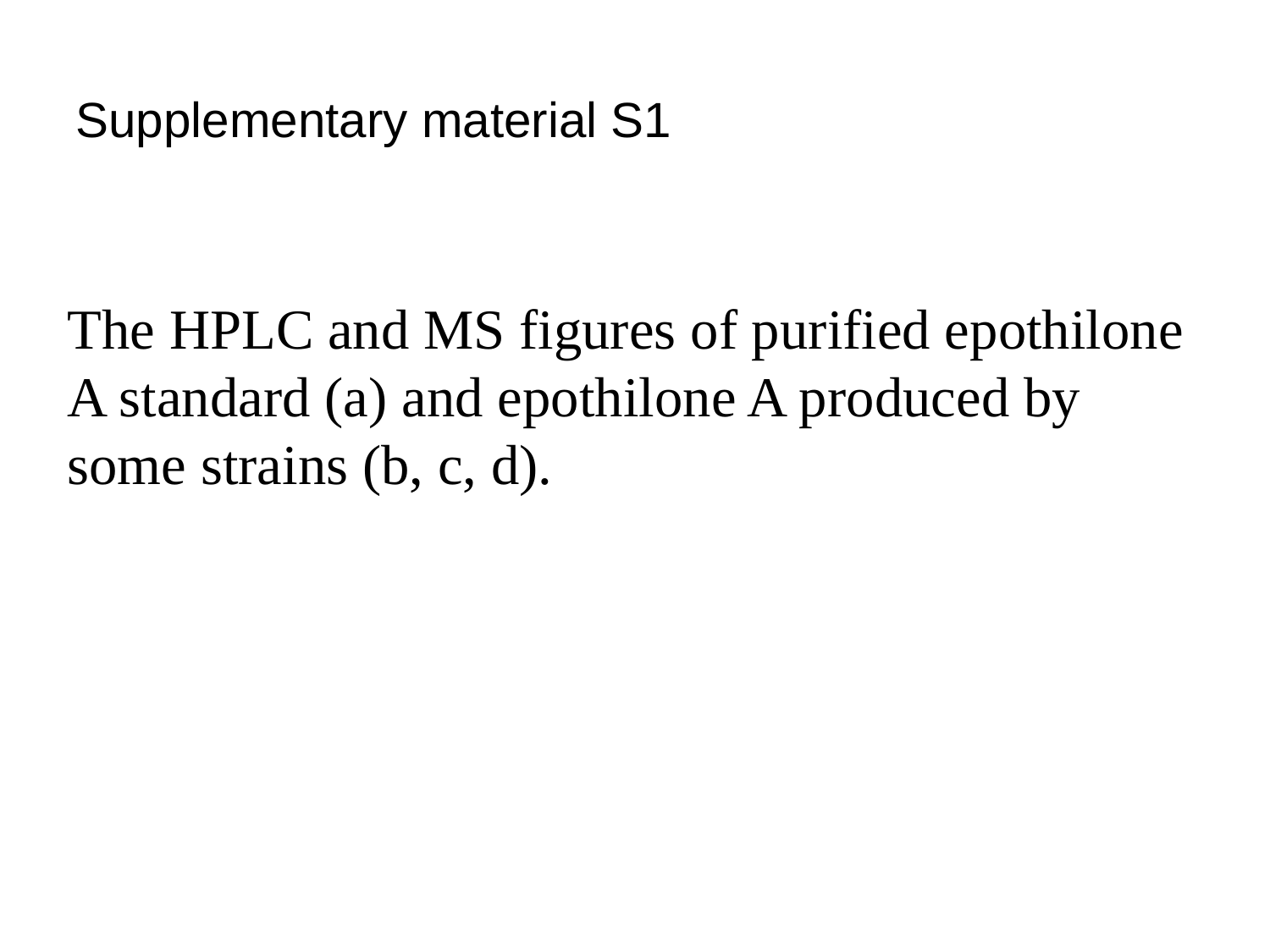

# Supplementary material S1
The HPLC and MS figures of purified epothilone A standard (a) and epothilone A produced by some strains (b, c, d).

## Slide 2
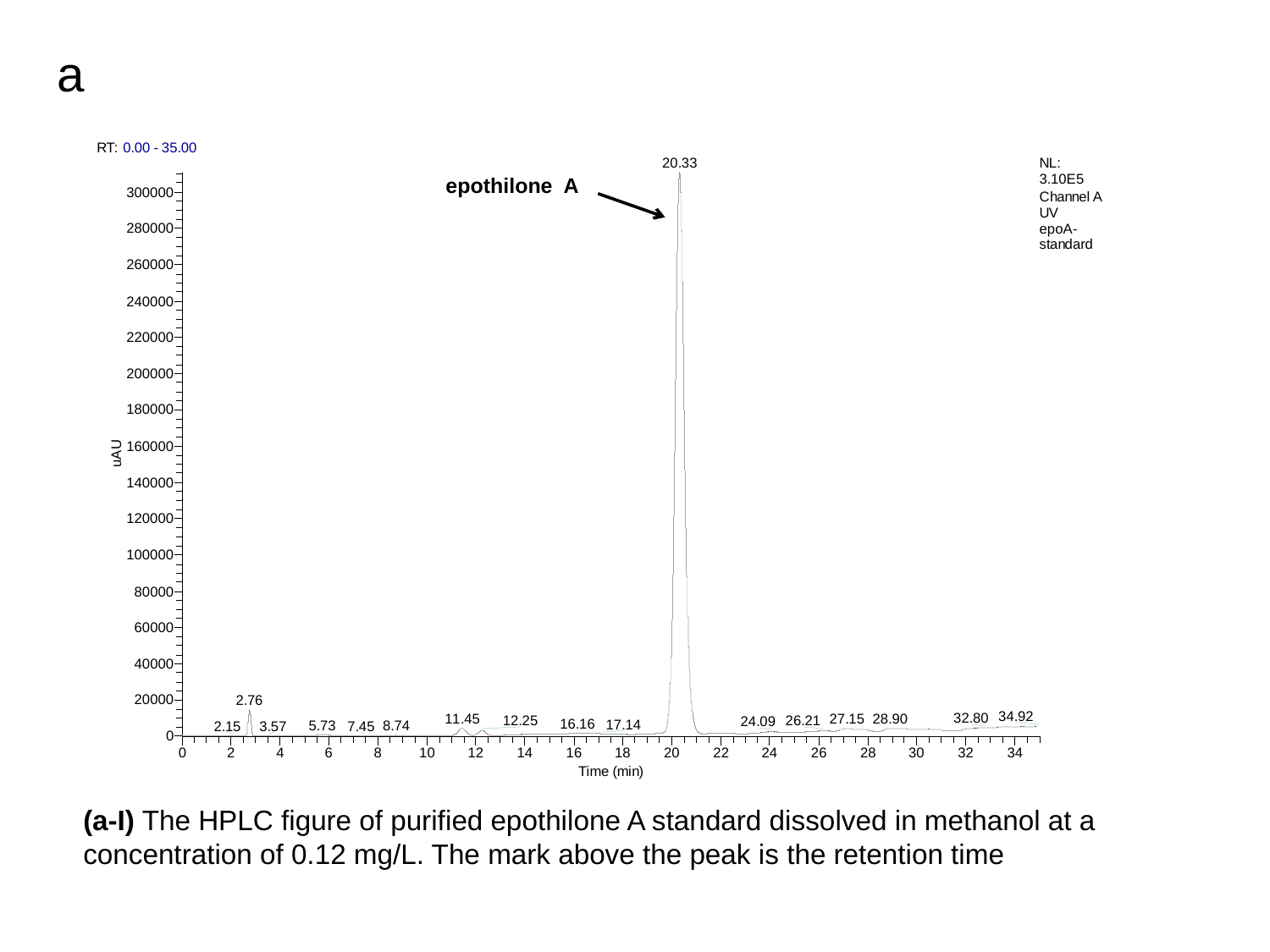

# a
epothilone A
(a-I) The HPLC figure of purified epothilone A standard dissolved in methanol at a concentration of 0.12 mg/L. The mark above the peak is the retention time

## Slide 3
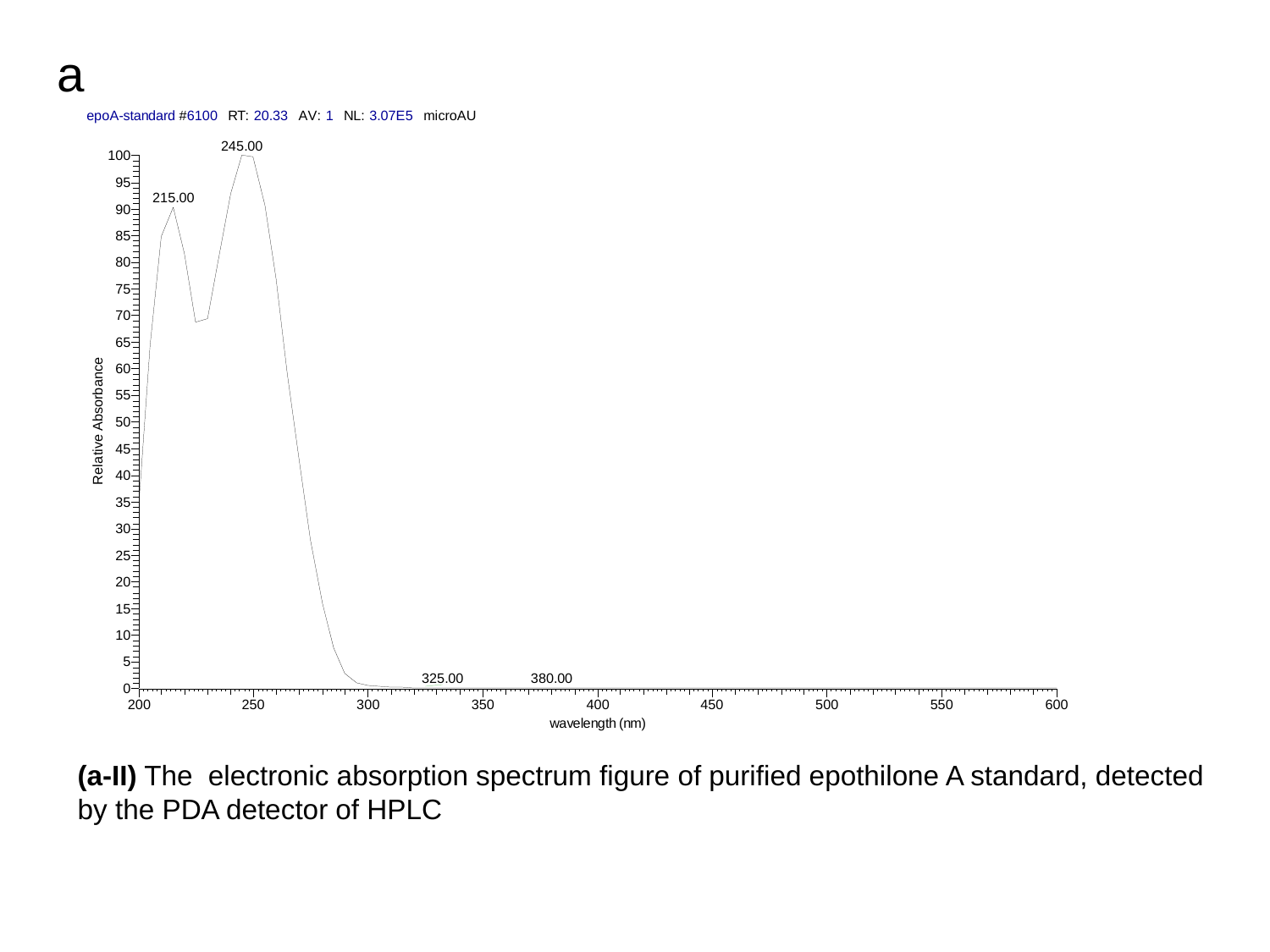

# a
(a-II) The electronic absorption spectrum figure of purified epothilone A standard, detected by the PDA detector of HPLC

## Slide 4
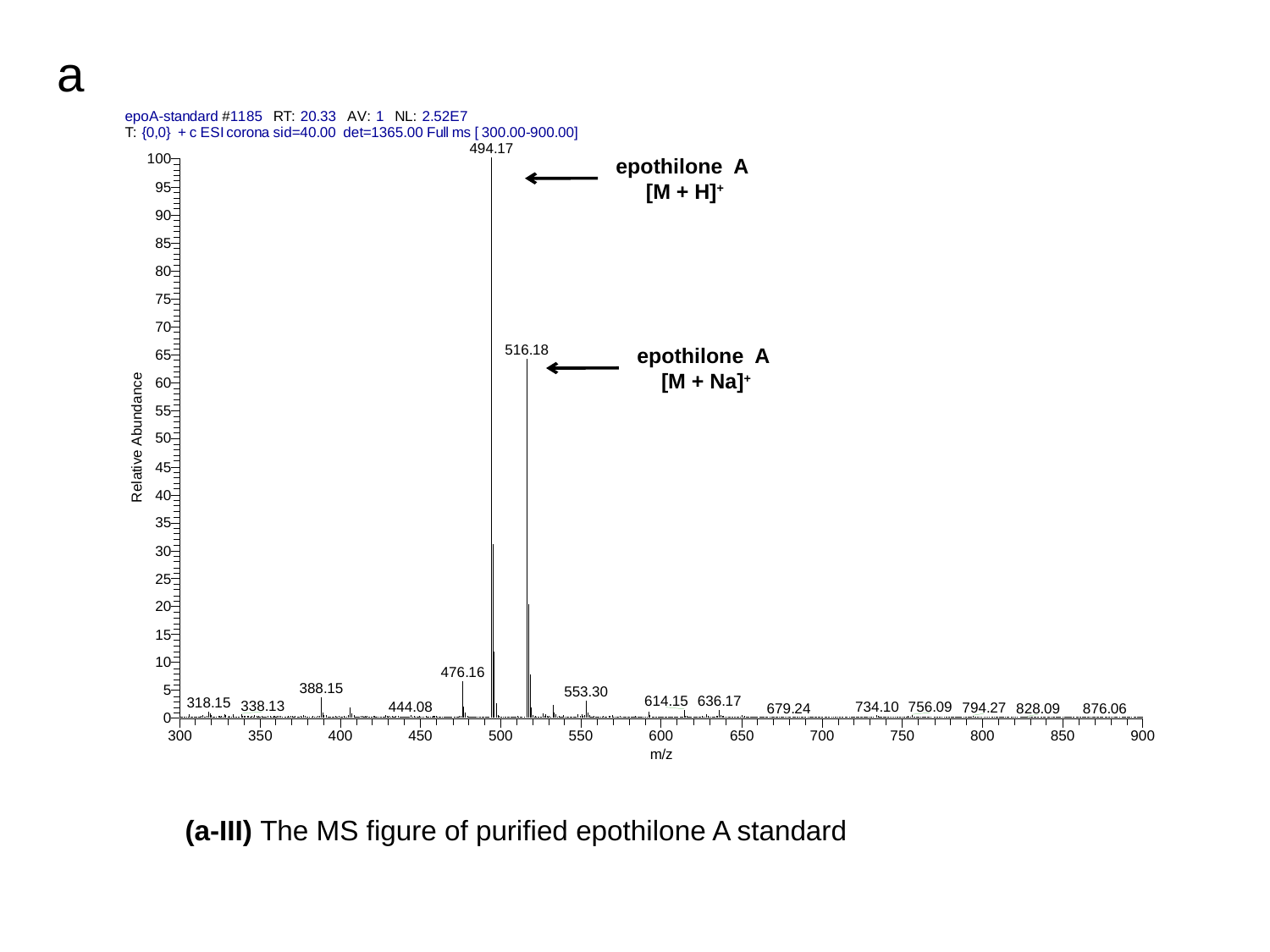

# a
epothilone A
[M + H]+
epothilone A
[M + Na]+
 (a-III) The MS figure of purified epothilone A standard

## Slide 5
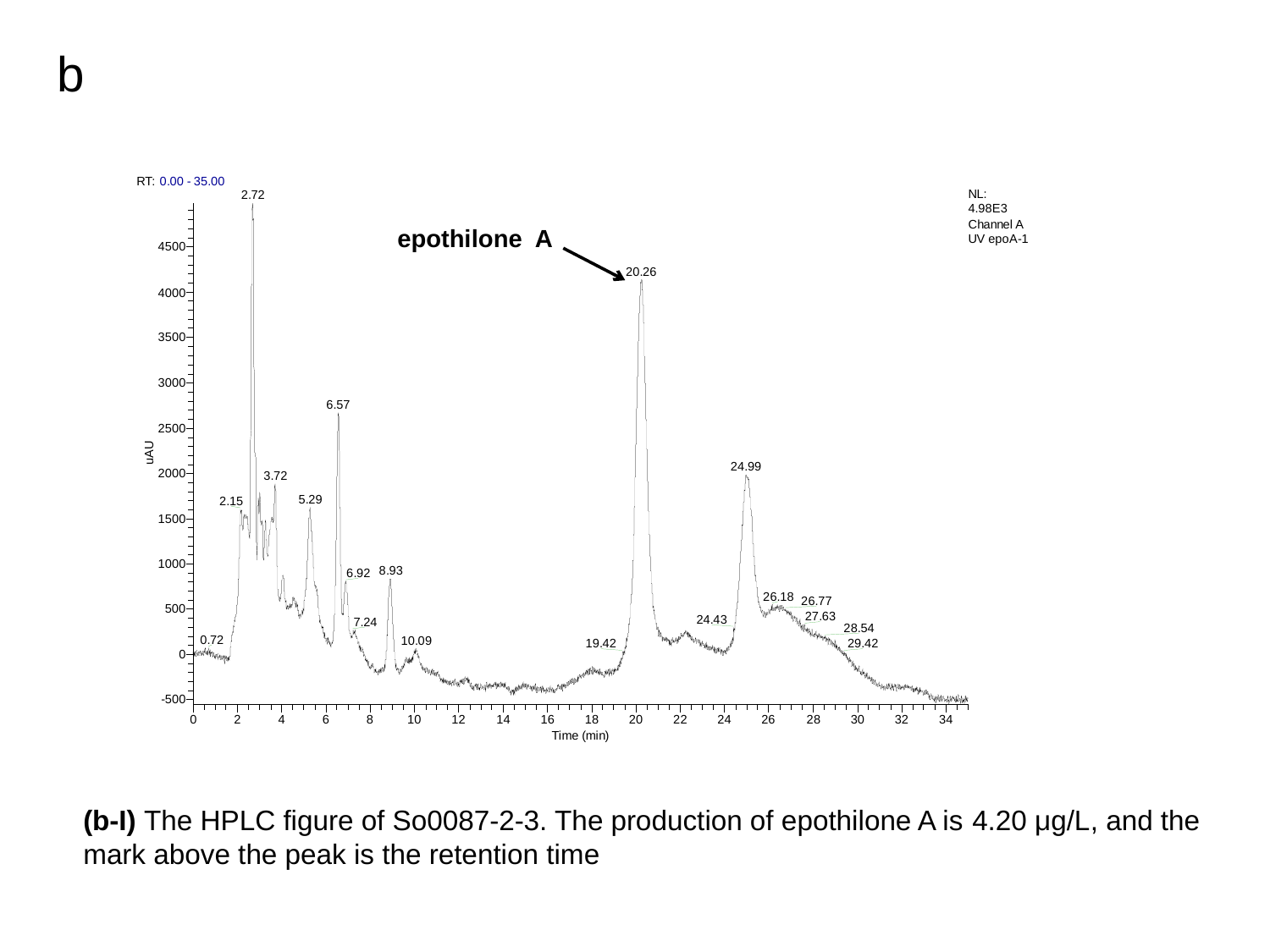

b
epothilone A
(b-I) The HPLC figure of So0087-2-3. The production of epothilone A is 4.20 μg/L, and the mark above the peak is the retention time

## Slide 6
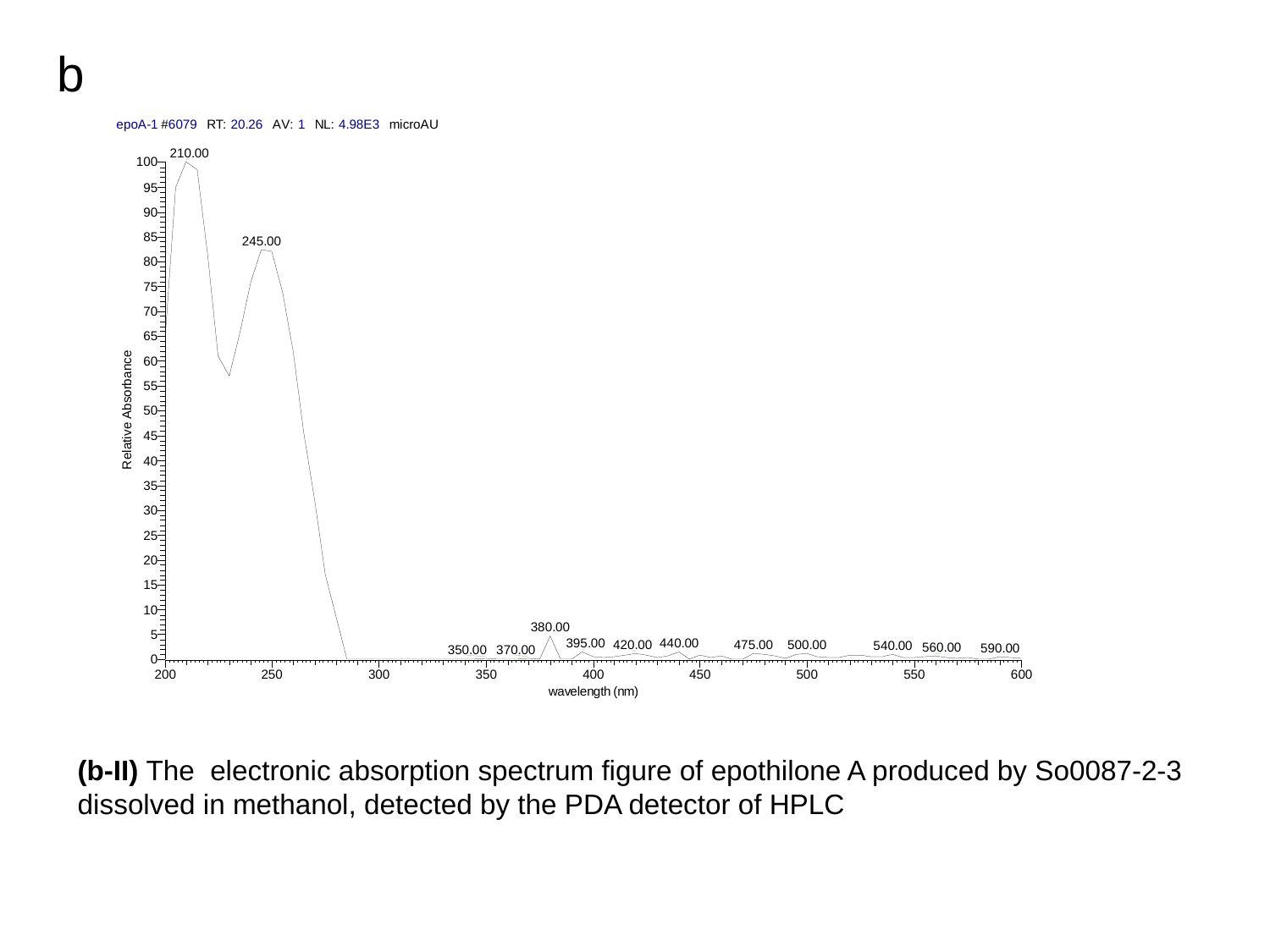

b
(b-II) The electronic absorption spectrum figure of epothilone A produced by So0087-2-3 dissolved in methanol, detected by the PDA detector of HPLC

## Slide 7
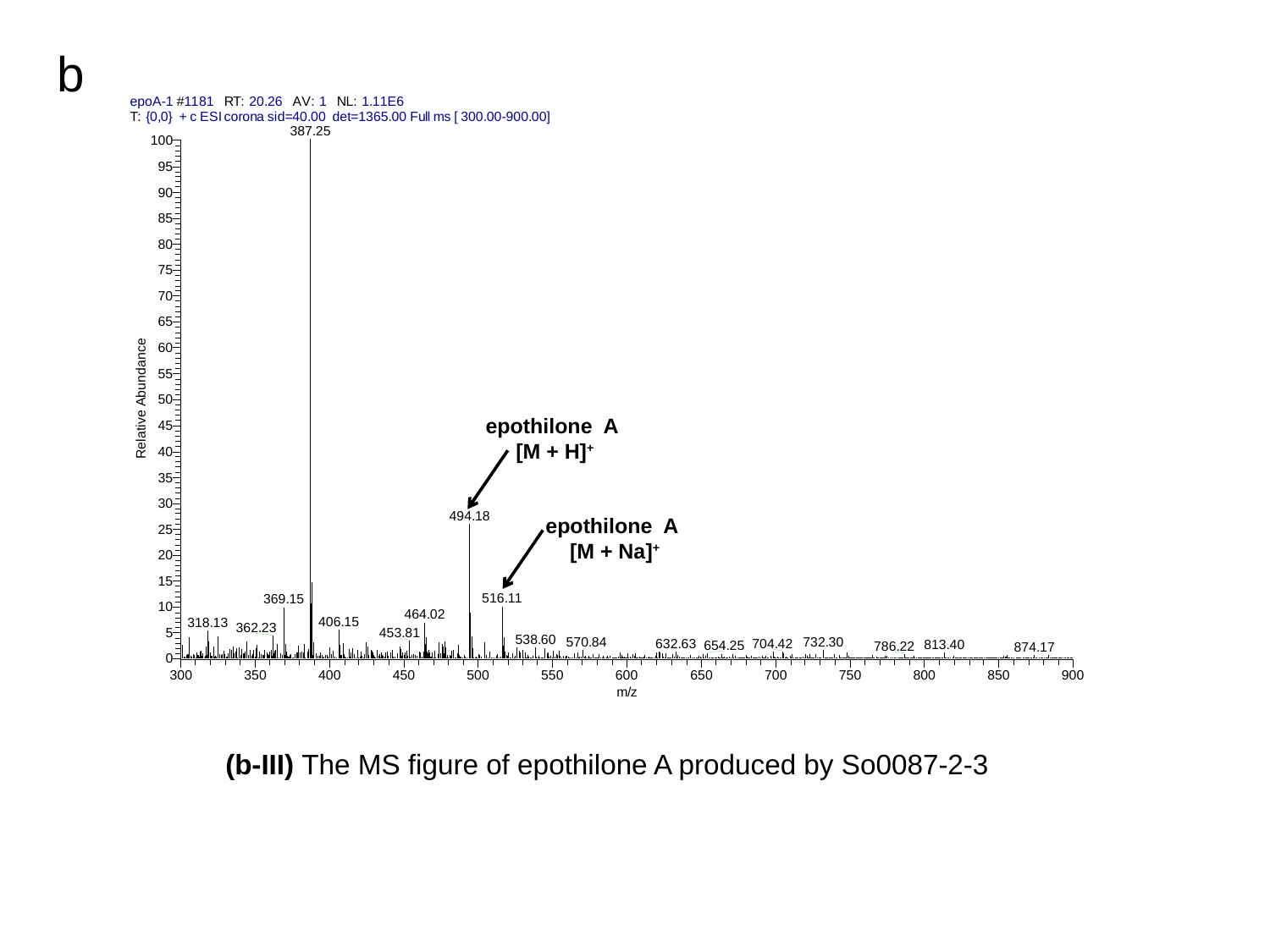

b
epothilone A
[M + H]+
epothilone A
[M + Na]+
 (b-III) The MS figure of epothilone A produced by So0087-2-3

## Slide 8
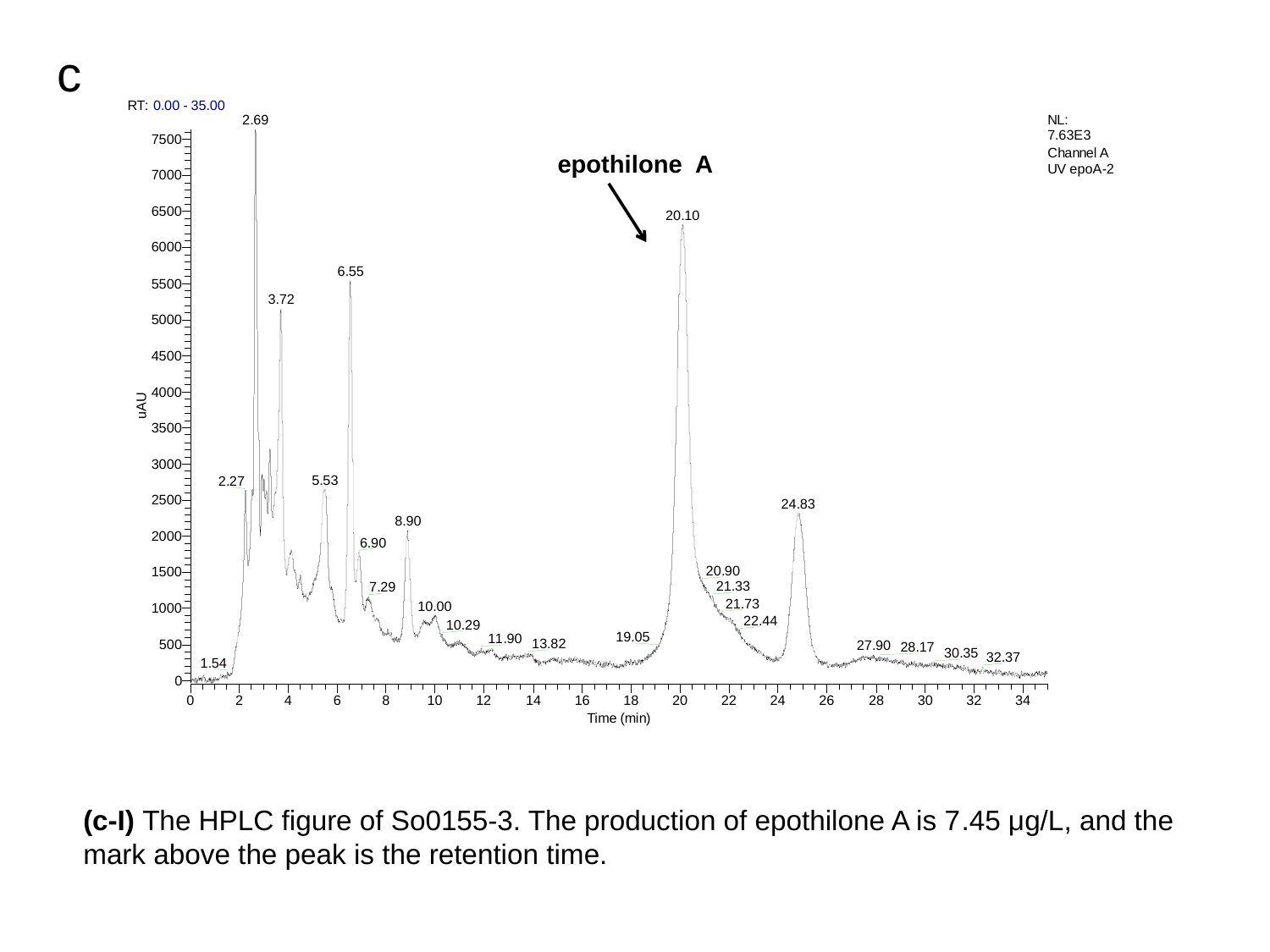

c
epothilone A
(c-I) The HPLC figure of So0155-3. The production of epothilone A is 7.45 μg/L, and the mark above the peak is the retention time.

## Slide 9
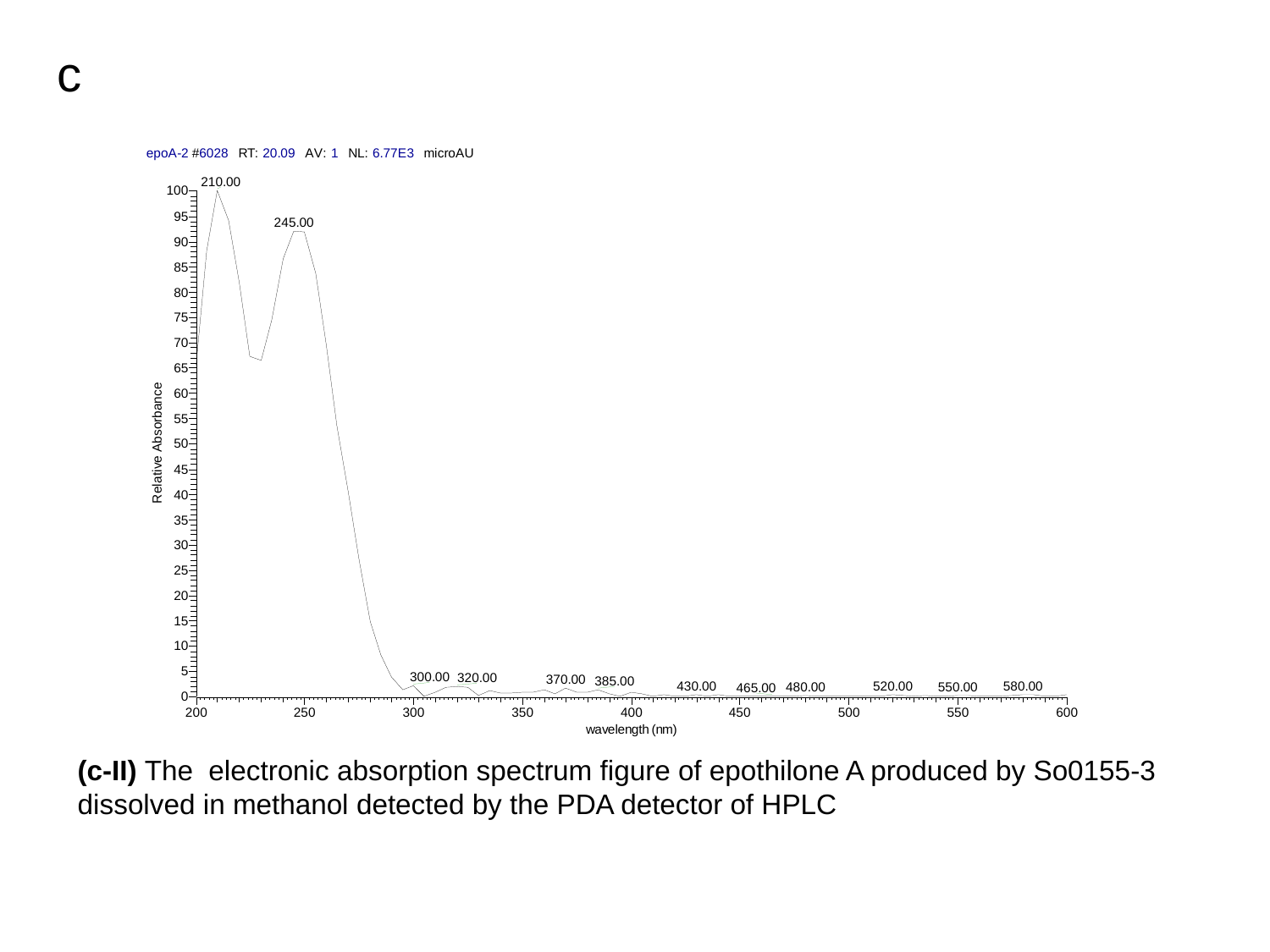

c
(c-II) The electronic absorption spectrum figure of epothilone A produced by So0155-3 dissolved in methanol detected by the PDA detector of HPLC

## Slide 10
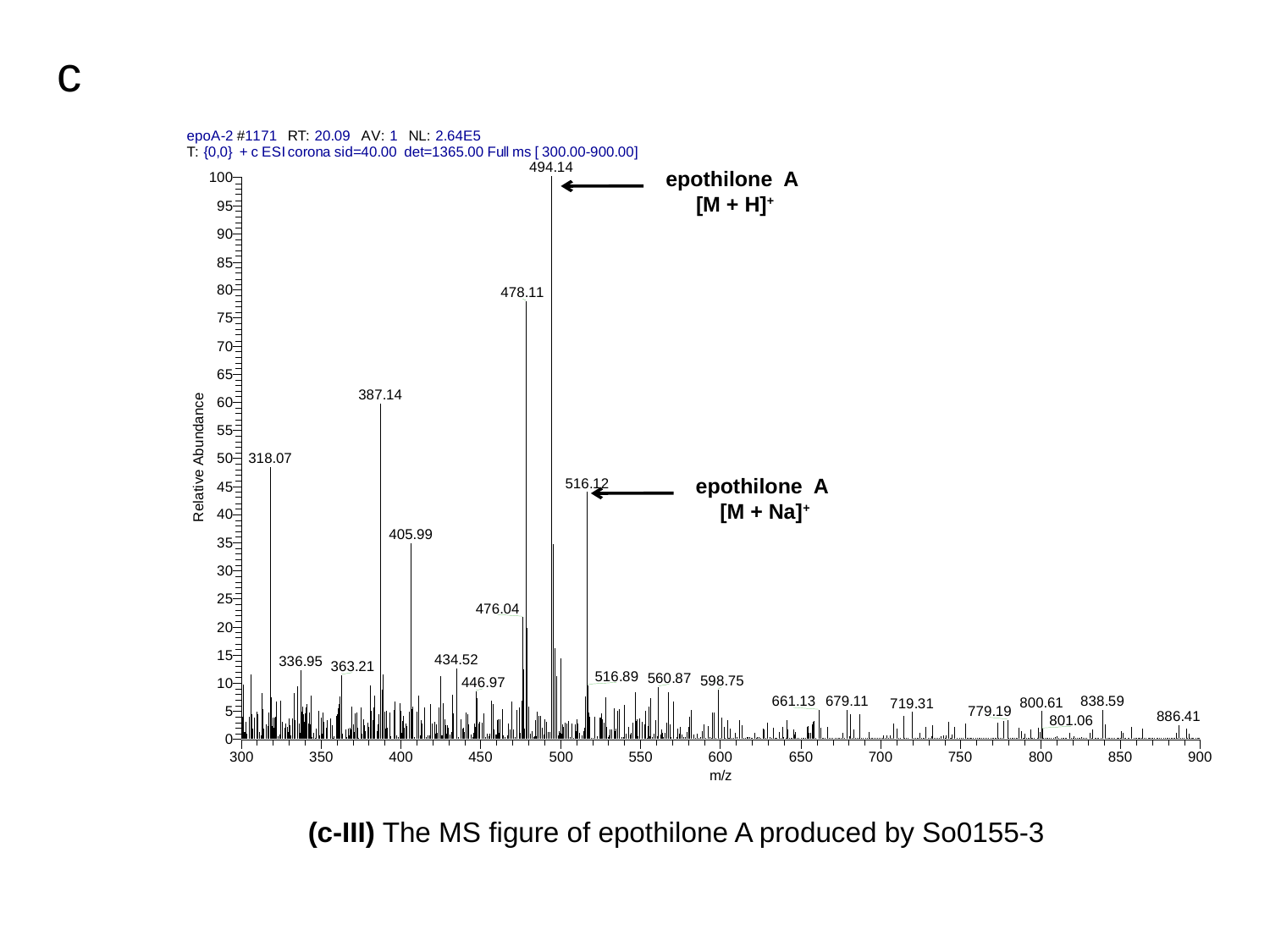

c
epothilone A
[M + H]+
epothilone A
[M + Na]+
 (c-III) The MS figure of epothilone A produced by So0155-3

## Slide 11
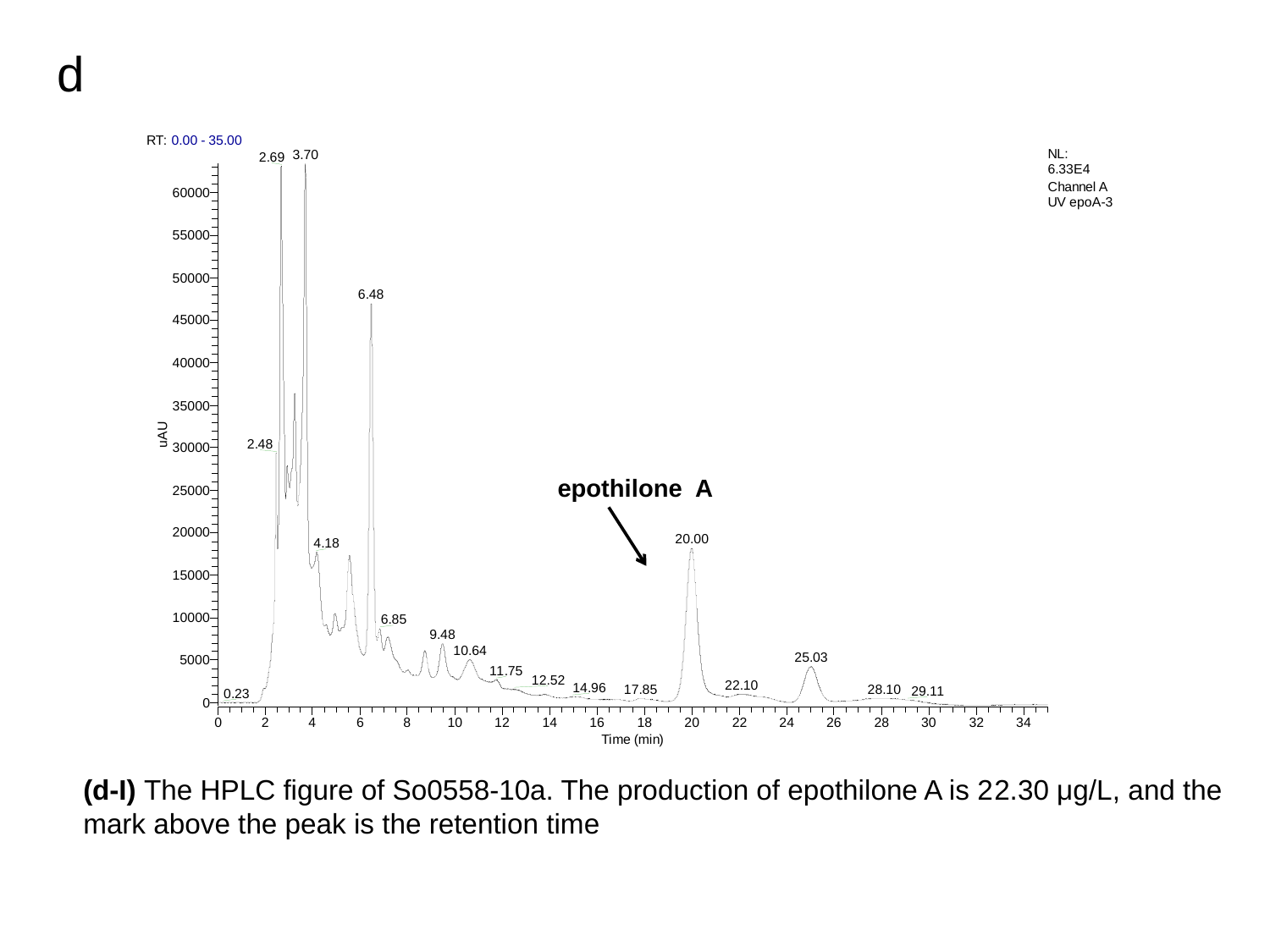

d
epothilone A
(d-I) The HPLC figure of So0558-10a. The production of epothilone A is 22.30 μg/L, and the mark above the peak is the retention time

## Slide 12
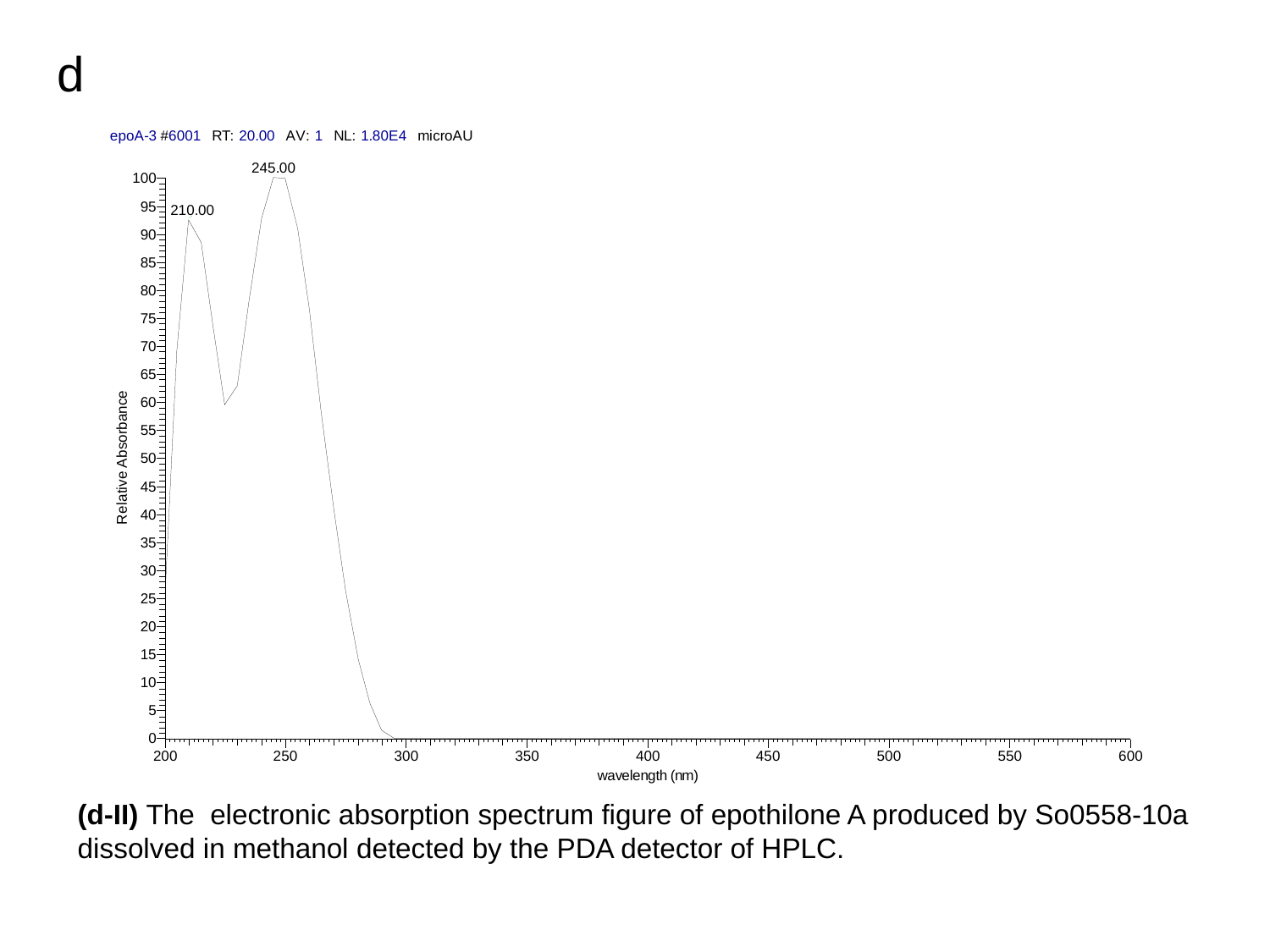

d
(d-II) The electronic absorption spectrum figure of epothilone A produced by So0558-10a dissolved in methanol detected by the PDA detector of HPLC.

## Slide 13
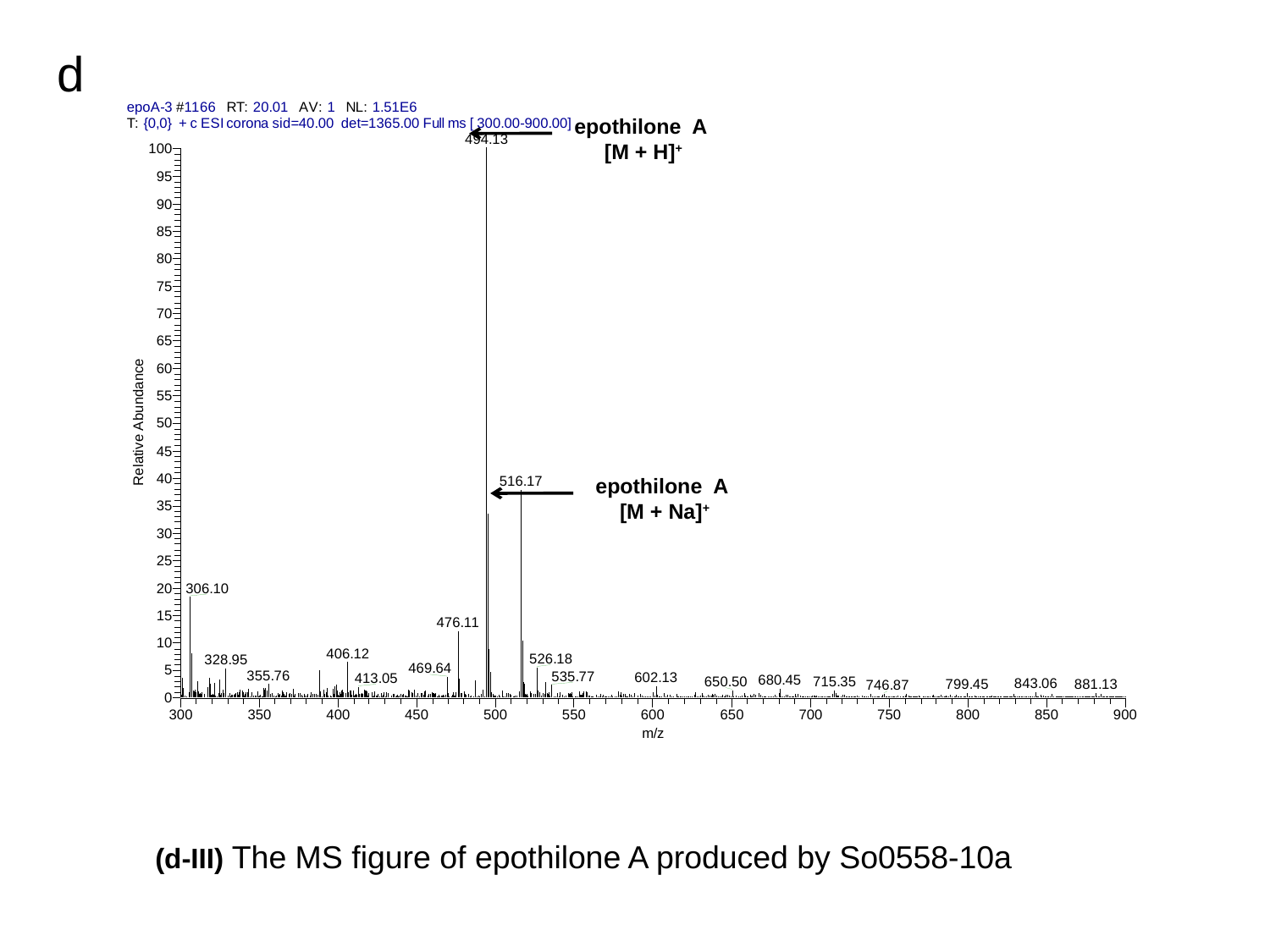

d
epothilone A
[M + H]+
epothilone A
[M + Na]+
 (d-III) The MS figure of epothilone A produced by So0558-10a
