# Supplementary figures and images for: Diversity of epothilone producers among Sorangium strains in producer-positive soil habitats
Source: Microb Biotechnol. 2013 Dec 6;7(2):130–41. doi: 10.1111/1751-7915.12103 (PMC3937717; doi:10.1111/1751-7915.12103)

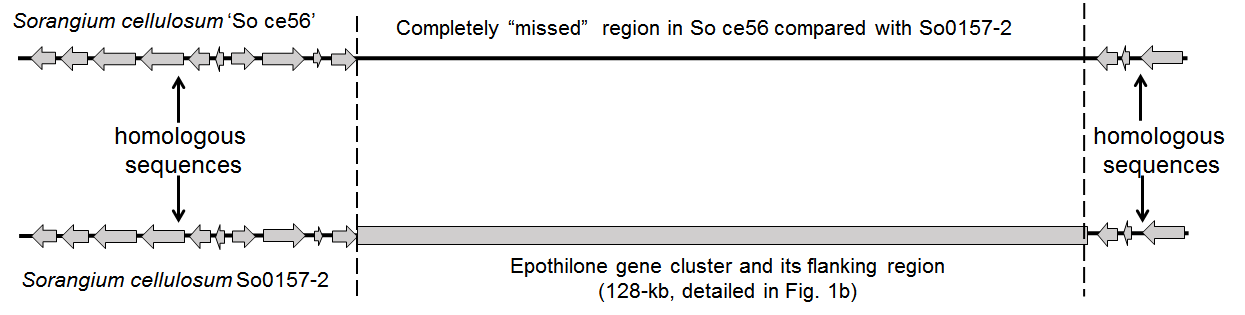

Supplement: Fig S1 — Phylogenetic analysis of the protein sequences of AT domains retrieved from sequenced myxobacterial genomes. Some non-myxobacterial AT sequences were taken as reference. AT domains from epothilone biosynthesis clusters were compressed into two red triangles for easy tracking. The trees were constructed in a cycle using the MEGA 5.05 programme. Bootstrap support was based on 1000 replicates. One unit along the bar is equivalent to 10 nucleotides change per 100 bp. The outermost cycle was added after construction. Beside S. cellulosum So0157-2 and So ce56, other myxobacterial strains used in AT domains extracting are Anaeromyxobacter dehalogenans 2CP-1, Anaeromyxobacter dehalogenans 2CP-C, Anaeromyxobacter sp. Fw109, Anaeromyxobacter sp. K, Haliangium ochraceum dsm14365, Myxococcus fulvus HW-1, Myxococcus xanthus DK1622, Stigmatella aurantiaca dw4/3. The two numbers linked by ‘...’; showed the positions of the AT sequences in the genome. [file mbt20007-0130-sd2.tif]

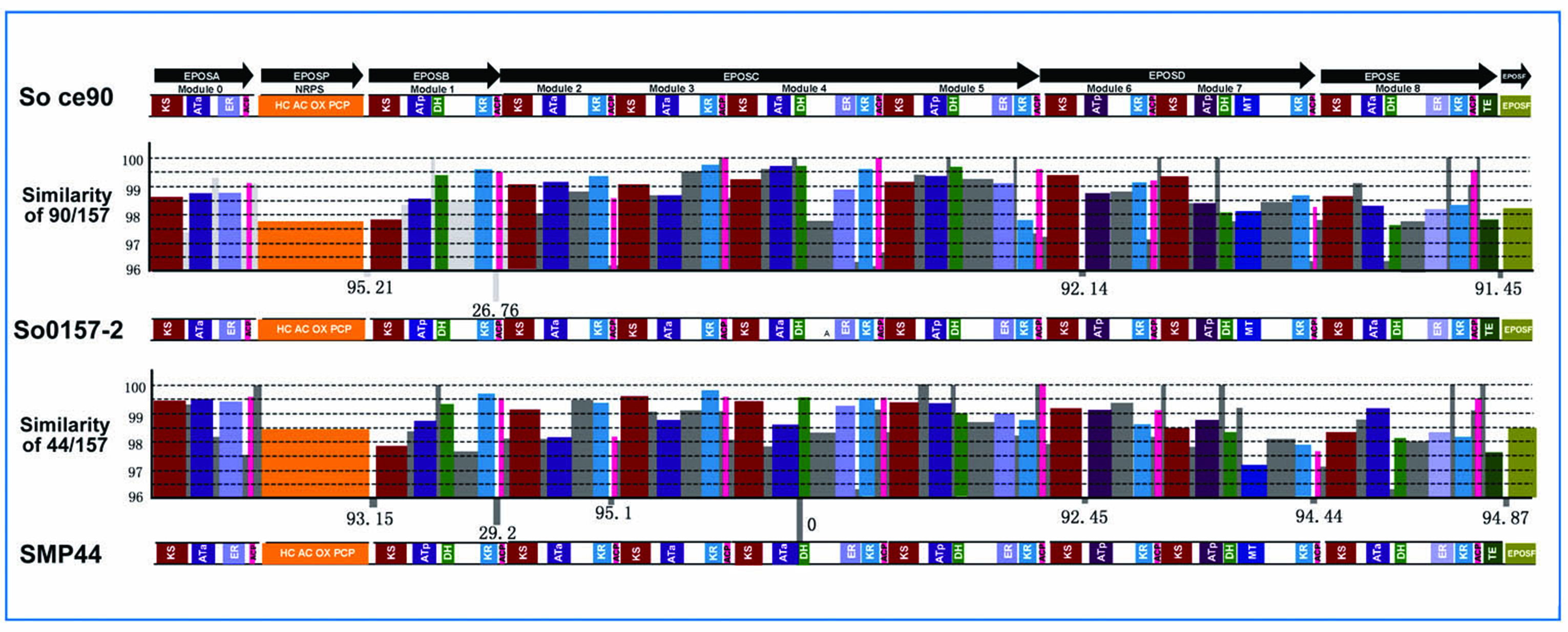

Supplement: Fig S2 — The upstream and downstream region of the ‘128 kb length epothilone gene cluster and its flanking region’ in So 0157-2, when compared with So ce56. The biosynthesis gene cluster for epothilones and its flanking regions were extracted from the completely sequenced genome of the So0157-2 strain (Han et al., 2013). The open reading frames (ORFs) were predicted using Glimmer 3.02 (Delcher et al., 2007) and GeneMark.hmm (Borodovsky and Lomsadze, 2011). All ORFs larger than 50 amino acid residues were blasted against the NR database of the NCBI with a cut-off of 1 E–5. The GC content was calculated by GEECEE of the EMBOSS programme (Rice et al., 2000). [file mbt20007-0130-sd3.tif]

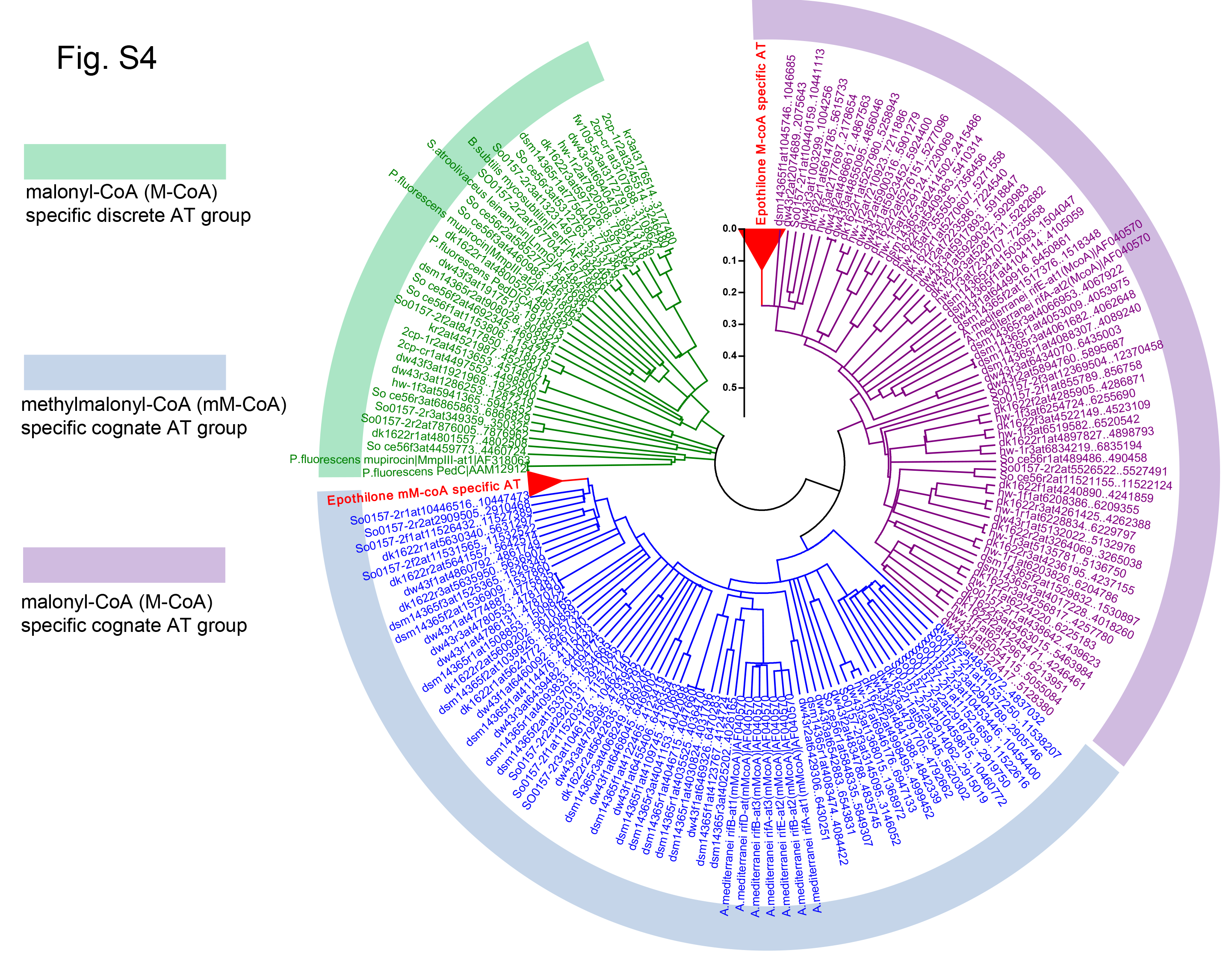

Supplement: Fig S3 — Similarity of the epothilone biosynthesis gene clusters (about 56 kb length) from three different Sorangium strains So ce 90 (AF210843), SMP44 (AF217189) and So0157-2 (CP003969). Each domain of the three whole gene clusters was homologous with an average of 98.5% identity, indicated by colourful histograms. The similarities of the intergenic spaces were also shown by grey histograms. [file mbt20007-0130-sd4.tif]
